# New Chimeric Antigen Receptor Design for Solid Tumors

**DOI:** 10.3389/fimmu.2017.01934

**Published:** 2017-12-22

**Authors:** Yuedi Wang, Feifei Luo, Jiao Yang, Chujun Zhao, Yiwei Chu

**Affiliations:** ^1^Department of Immunology, School of Basic Medical Sciences, Fudan University, Shanghai, China; ^2^Biotherapy Research Center, Fudan University, Shanghai, China; ^3^Department of Digestive Diseases, Huashan Hospital, Fudan University, Shanghai, China; ^4^Northfield Mount Hermon School, Mount Hermon, MA, United States

**Keywords:** chimeric antigen receptor T-cell, immunotherapy, solid tumor, adoptive T-cell therapy, tumor microenvironment

## Abstract

In recent years, chimeric antigen receptor (CAR) T-cell therapy has become popular in immunotherapy, particularly after its tremendous success in the treatment of lineage-restricted hematologic cancers. However, the application of CAR T-cell therapy for solid tumors has not reached its full potential because of the lack of specific tumor antigens and inhibitory factors in suppressive tumor microenvironment (TME) (e.g., programmed death ligand-1, myeloid-derived suppressor cells, and transforming growth factor-β). In this review, we include some limitations in CAR design, such as tumor heterogeneity, indefinite spatial distance between CAR T-cell and its target cell, and suppressive TME. We also summarize some new approaches to overcome these hurdles, including targeting neoantigens and/or multiple antigens at once and depleting some inhibitory factors.

## Introduction

Chimeric antigen receptor (CAR) design is based on the signal transduction of T-cell activation (1). The T-cell receptor (TCR) detects antigens presented by antigen-presenting cells (APCs) in the form of the major histocompatibility complex (MHC)–antigen peptide complex (2). TCR binding to the MHC–antigen peptide complex induces a cascade of intracellular events as follows: phosphorylated TCR recruits intracellular second messengers to provide the first signal, and costimulatory molecules (CD28, CD27, CD134, CD137, or ICOS) at the T-cell surface bind to their corresponding receptors (CD80, CD86, CD137L, or ICOSL) on APCs, which further provides the second signal (3). Eventually, T-cells are primed and activated, which subsequently secrete perforin, granzyme, and cytokines, including interleukin 2 (IL-2) and interferon γ (IFN-γ), to defend infection by inducing the apoptosis of target cells.

However, normal T-cells do not efficiently recognize tumors because of the absence of MHC expression and weak immunogenicity of tumors. Investigators first developed chimeric immune receptors in mid-1980s. In 1993, Eshhar et al. modified the T-cell expressing CARs in melanoma treatments, which overcame the issue of MHC restriction and weak immunogenicity (4). Generally, CARs comprise three domains: an extracellular single-chain antibody fragment (scFv), which serves as a target moiety that redirects T-cells to tumor cells by specifically binding to tumor-associated antigens (TAAs); a transmembrane domain and an endodomain, which is often the signal transduction domain comprising a CD3ζ chain and costimulatory factors such as CD28 and 4-1BB (CD137) (5, 6). According to different intracellular domains, CARs are divided into three generations. The intracellular domain of the first generation contains only a CD3ζ chain; the second contains a CD3ζ chain and a costimulatory molecule [CD28, 4-1BB, CD134 (OX40), or ICOS]; and the third contains a CD3ζ chain and two or more different costimulatory molecules (7, 8). Zhang et al. compared CD28 with 4-1BB as a costimulant and demonstrated that 4-1BB was essential for expanding memory CD8^+^ T-cells and was superior to CD28 in costimulating the generation of CD8^+^ cytotoxic lymphocytes (9). Therefore, using 4-1BB as a costimulation factor in CAR designs may hold promise for ameliorating exhaustion and improving the effectiveness of CAR T-cell.

**Figure 1:**
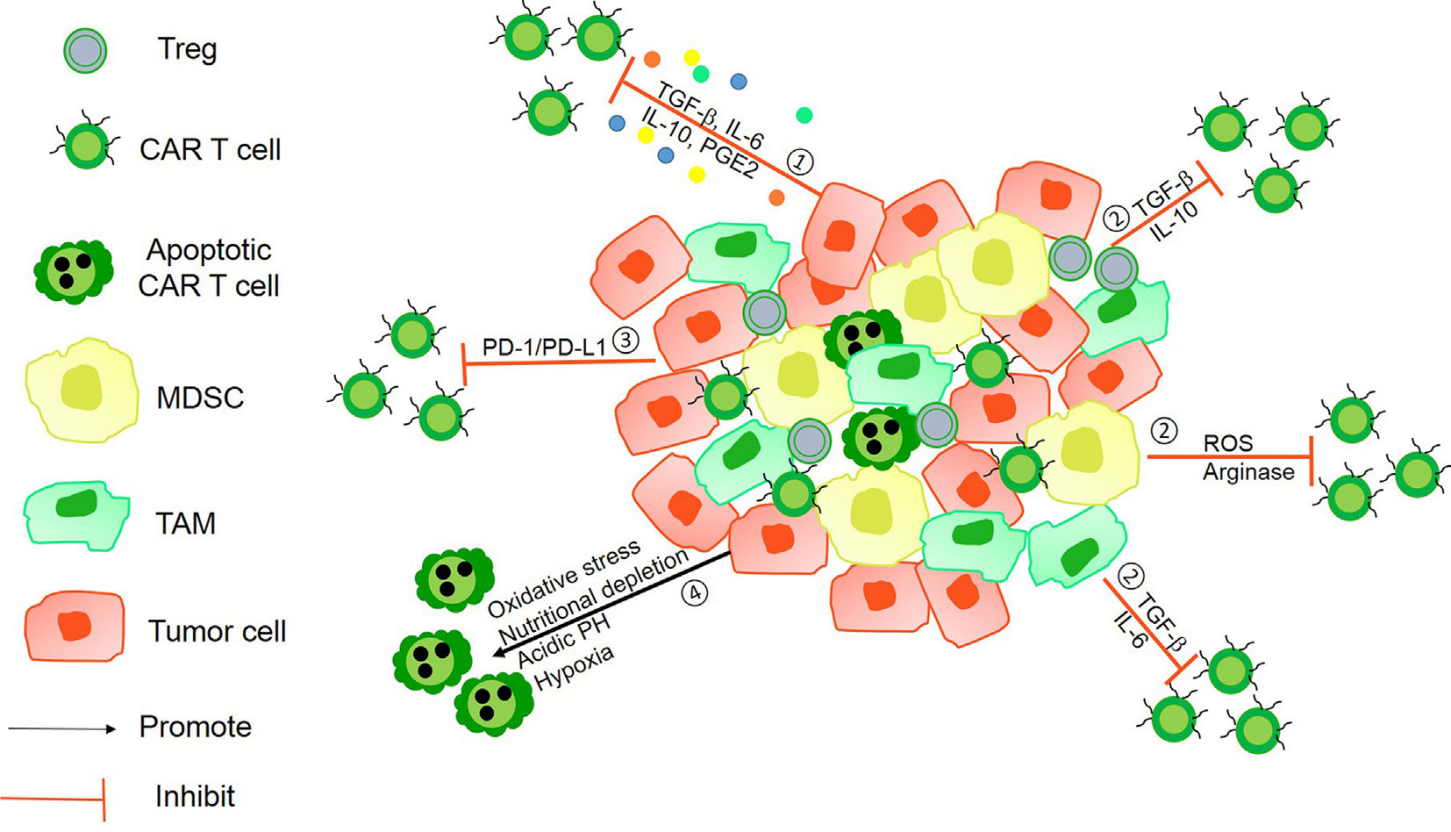
The immunosuppressive mechanisms of tumor microenvironment (TME). ① Tumor-derived soluble factors such as prostaglandin E2 (PGE2) and cytokines such as transforming growth factor-β (TGF-β), IL-6, and IL-10 inhibit chimeric antigen receptor (CAR) T-cells vitality. ② The presence of immunosuppressive immune cells, namely myeloid-derived suppressor cells (MDSCs), Tregs, tumor-associated microphages (TAMs), or neutrophils (TANs), suppress T-cell function *via* Arg-I, ROS generation, and some soluble inhibitory factors. ③ Tumor cells can utilize the intrinsic negative regulatory mechanisms of T-cells by upregulating surface inhibitory receptors such as programmed death ligand-1 (PD-L1)/PD-L2. ④ The hostile TME makes it difficult for CAR T-cells to survive because of hypoxia, oxidative stress, acidic pH, and nutritional depletion.

There have been an increasing number of CAR T-cell clinical trials for solid tumors because of their unprecedented efficacy for non-solid tumors, particularly anti-CD19 CAR T-cell (Table 1). An important reason for the success of CAR T-cell therapy in leukemia is that because non-solid tumor cells circulate within the blood and lymphatic system, they are more likely to meet adoptive CAR T-cells and induce them into killing activity. However, in solid tumors, it is particularly difficult for CAR T-cells to migrate into tumor sites because of several obstacle layers, such as the extracellular matrix, and the lack of chemokines, which are frequently mismatched with receptors in solid tumors (10). Even if a few CAR T-cells successfully infiltrate the tumor sites, they may become inactivated because of the suppressive tumor microenvironment (TME) (11–14) (Figure 1). Another important reason is that it is extremely difficult to find a specific TAA, such as CD19, in B-cell acute lymphoblastic leukemia (B-ALL) for solid tumors (15). Therefore, CAR T-cells require novel additional modifications for an enhanced antitumor efficiency.

**Table 1:** Summary of CAR T cell therapy for solid tumor.

CARs design	Gene transfer vehicle	Malignancy	Trial design	Outcome	ClinicalTrials.gov Identifier	Reference
Anti-EGFRvIII scFv-4-1BB/CD28-CD3ζ	Lentivirus	Glioblastoma	Phase 1	Recruiting	NCT02209376	(16, 17)
Anti-mesothelin scFv-4-1BB-CD3ζ	mRNA electroporation	Pancreatic cancers, mesotheliomas, ovarian cancers, lung cancers	Phase 1	Active, not recruiting	NCT01355965	(18)
Anti-glypican-3 scFv-CD28-4-1BB-CD3ζ	Retrovirus	HCC, MRT, hepatoblastoma, embryonal sarcoma, lung cancers	Early phase clinical trials	Not recruiting	NCT02905188	(19–21)
Anti-ErbB2 scFv-4-1BB-CD3ζ	mRNA electroporation, lentiviral transduction	Lung cancer, ovarian cancer, breast cancer	Preclinical trails	–	–	(22)
Anti-PSMA scFv-CD3ζ + IL-2	Retrovirus	Prostate cancer	Phase 1	2/5 PR	NCT01929239	(23)
Anti-HER2 scFv-CD28-CD3ζ	Retrovirus	Breast cancer, sarcoma	Phase 1/2	13/19 PD 2/19 NE 4/19 SD	NCT00902044	(24)
Anti-EGFR scFv-CD137-CD3ζ	Lentivirus	Non-small-cell lung cancer	Phase 1	5/11 SD 4/11 PD 2/11 PR	NCT01869166	(25–27)
Anti-MUC1 scFv-CD28-OX40-CD3ζ	Retrovirus	HCC, non-small lung cancer, triple-negative breast cancer	Preclinical trails	–	–	(28, 29)
Anti-CEA scFv-CD28-CD3ζ	Retrovirus	Liver metastases	Phase 1	1/6 SD 5/6 PD	NCT01373047	(30, 31)
IL13Rα-4-1BB-CD3ζ	Retrovirus	Glioblastoma	Phase 1	1/1 CR	NCT02208362	(32, 33)

*PD, progressive disease; PR, partial response; SD, stable disease; NE, not evaluable; CR, complete remission; CAR, chimeric antigen receptor; scFv, single-chain antibody fragment; PSMA, prostate-specific membrane antigen; IL-2, interleukin 2*.

## Target Antigen Specificity

Reports of clinical trials of CAR T-cell therapy for solid tumors have shown that most CAR T-cell therapies are stuck at the “on-target, off-tumor” stage (34, 35). The ideal TAAs need to be specifically expressed on tumor cells; however, several TAAs are also expressed on normal cells. For instance, mesothelin is not only overexpressed on mesothelioma but also expressed on peritoneal, pleural, and pericardial surfaces (36). Moreover, most tumor cells remove their immunogenic epitopes of TAAs to escape the attack of the host’s immune system. Therefore, identifying specific and immunogenic tumor antigens is necessary for the treatment of solid tumor.

Investigators may design CARs targeting aberrantly modification of TAAs or tumor-specific oncogenic mutations such as truncated MUC1. For example, Posey et al. recently described a new CAR targeting aberrantly glycosylated tumor-associated cell membrane mucin (MUC1). In this study, they used a second-generation CAR with 4-1BB as a costimulatory molecule, and the binding domain was the scFv region of the high-affinity antibody (5E5) targeting truncated *O*-glycopeptide epitopes specifically presented on tumor tissues. Thus, these CAR T cells normally did not bind to glycosylated MUC1, but they specifically recognized the Tn glycoform of MUC1 on tumor cells in this case. This study also demonstrated that MUC1–CAR T-cell exhibited no cytotoxicity against normal human primary cells (37).

Investigators are also examining neoantigens specifically expressed on tumor cells (38). Neoantigens are antigens resulting on tumor cells from somatic mutations and are unique to each patient’s cancer, and these mutations may facilitate tumor growth and/or invasion. Using neoantigens as a target can minimize the risk of killing healthy tissue (39). Several investigators have proposed using next-generation sequencing combined with high-throughput immunological screening approaches to identify immunogenic mutations (40, 41). They separated normal cells from tumor cells obtained from patients and subsequently used whole-exome and transcriptome sequencing to identify somatic mutations, which could be presented by APCs, and activate an immune response (42). Verdegaal et al. found that T-cell mediated neoantigen immunoediting and the loss of expression of T-cell-recognized neoantigens may result in tumor resistance (43). Therefore, it is imperative to monitor the neoantigen landscape dynamics during adoptive T-cell therapy.

**Figure 2:**
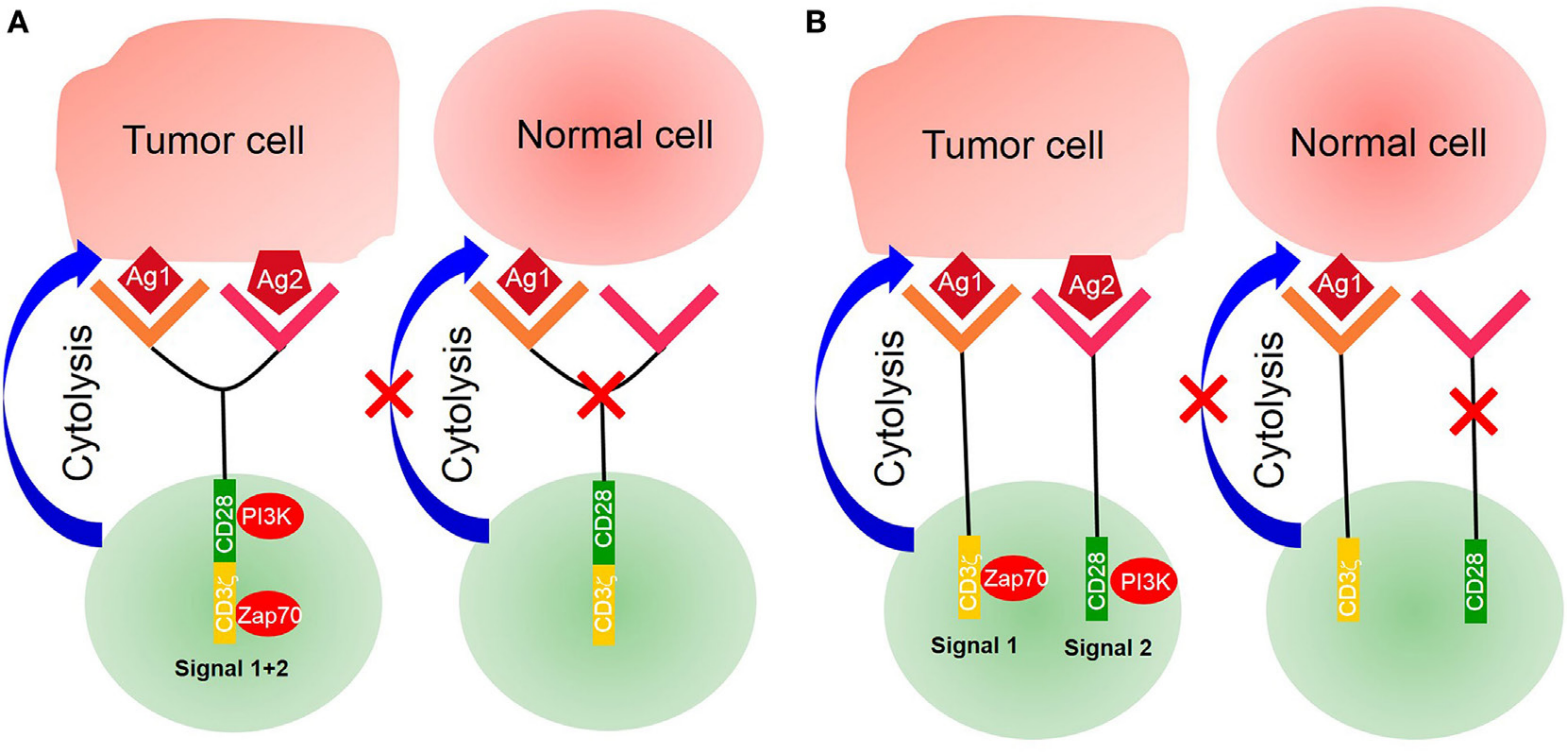
Tandem chimeric antigen receptor (CAR) T-cell. **(A)** The extracellular binding domain of CARs comprises two different tumor-associated antigen (TAA)-specific single-chain antibody fragments (scFvs) linked to the intracellular signaling domains derived from the CD3ζ chain and CD28 or CD137. CAR T-cell activated by two separate TAAs binding with TAA-specific scFv. **(B)** CD3ζ chain of CARs was designed with costimulatory factors. A single CAR structure contains two scFvs—one linked CD3ζ chain, providing the first signal, and another linked costimulatory factors, providing the second signal. Only activating double signals activate T-cells.

More recently, a new kind of CARs—tandem CARs—has been designed to express two antigen-binding domains; the tandem CAR T-cell is activated only when simultaneously recognizing two different antigens (Figure 2). CARs, engineered to simultaneously target two different antigens, are more specific and safe. For example, Hegde et al. developed a tandem CAR by joining an anti-human epidermal growth factor receptor-2 (HER2) scFv and an IL-13 receptor α2 (IL-13Rα2)-binding IL-13 mutant and used CD28 as a costimulatory factor and CD3ζ chain as a signal transduction domain. The tandem CAR T-cell showed the potential to bind with either HER2 or IL-13Rα2 and to protect against tumor cells. Compared with single CAR T-cell upon encountering HER2 or IL-13Rα2, the activation dynamics of these CAR T-cells were more sustained but not more exhaustible. In a murine glioblastoma model, the tandem CAR T-cells mitigated antigen escape displayed enhanced antitumor efficacy and improved animal survival (44).

Another tandem CAR has been designed to deliver two separate scFvs—one linked to a CD3ζ chain providing the first signal and another to a costimulatory molecule providing the second signal. The expression of target antigens alone is insufficient to trigger T-cell activation. Only two antigens that are simultaneously expressed on a target cell can activate a CAR T-cell and induce an antitumor function (45). For example, investigators presented an approach to render CAR T-cells specific for prostate tumors even in the absence of a truly tumor-restricted antigen. In their work, they used two prostate tumor antigens—prostate-specific membrane antigen (PSMA) and prostate stem cell antigen (PSCA)—and demonstrated that CAR T-cell destroyed tumor cells expressing both PSMA and PSCA. In a murine syngeneic model, it was also shown that CAR T-cells were activated and protected against tumors simultaneously expressing PSCA and PSMA (46, 47). However, under the pressure of antigen-specific T-cells, tumor cells generate new mutations with a loss of antigen expression accompanied by resistance. Investigators need to monitor the antigen landscape dynamics to enhance CAR T-cell therapy.

## Target Antigen Sensitivity

**Figure 3:**
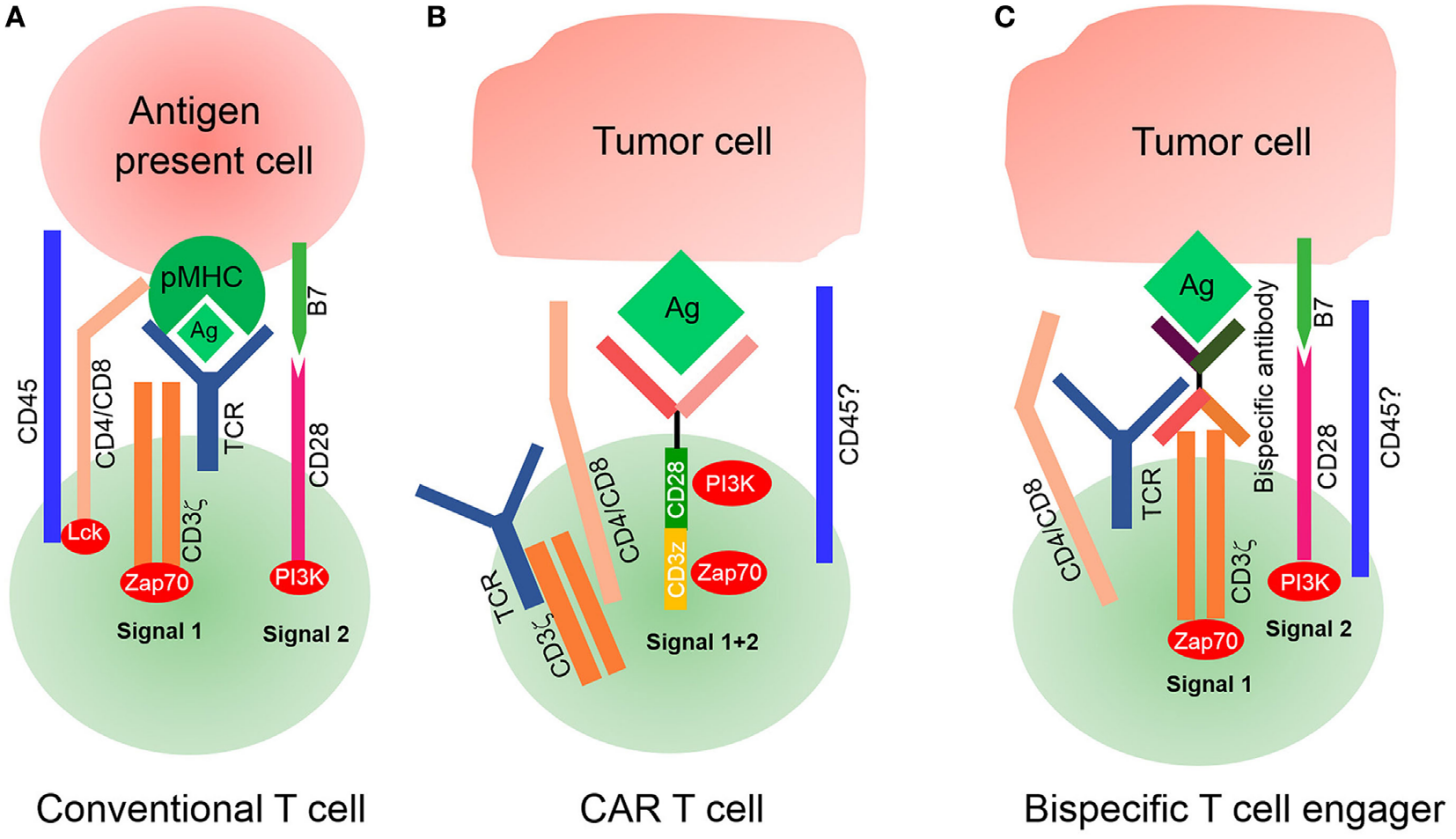
Signaling of conventional T-cell and chimeric antigen receptor (CAR) T-cell. **(A)** Conventional T-cell activation is initiated when T-cell receptor (TCR) interacts with pMHC for the formation of an immunological synapse. The spatial distance between T-cells and antigen-presenting cells (APCs) is approximately 15 nm, which physically excludes CD45 from the synapse because of its large ectodomain. CD4/CD8 molecules bind with major histocompatibility complex (MHC)I/II to recruit lymphocyte-specific kinase (Lck) phosphorylated by CD45, which then activates Zap70 to provide signal 1. Costimulatory molecules such as CD28 bind with their ligands on APCs to deliver signal 2 for complete T-cell activation. **(B)** Modified CAR T-cells recognize tumor cells by their tumor-associated antigens (TAAs) in a non-MHC restrictive manner. The spatial distance between CAR T-cells and target tumor cells is unknown, nor it is known whether this distance is small enough to physically exclude phosphatase CD45 from the synapse. It is also unknown whether CARs interact with endogenous TCR/CD3ζ or CD4/CD8 coreceptors. **(C)** Bispecific T-cell engagers (BiTEs) can secrete bispecific antibodies, one of which can recognize TAAs and another ligates with the intrinsic TCR–CD3 complex, but it is unknown if CD4/CD8 T-cells participate because of deficient MHC expression on tumor cells. Endogenous TCR/CD3ζ delivered signal 1 upon BiTEs ligation with target-expressing cells by secreting bispecific antibodies, and signal 2 is delivered by an intrinsic costimulatory molecule on BiTEs and its receptor lies on tumor cells. The spatial distance between BiTEs and tumor cells is also uncontrollable; therefore, it is also unknown if CD45 is excluded from the synapse.

Sensitivity is another challenge of CAR T-cell therapy for the treatment of solid tumors. The spatial distance between T-cells and their target cells also plays a key role in T-cell activation and signal transduction. It is essential for T-cell activation that immune receptor tyrosine-based activation motifs were phosphorylated by the lymphocyte-specific kinase (Lck) of the Src family (48). However, Lck is originally inhibited on T-cells so that it does not exhibit phosphorylation activity. It is activated by the protein tyrosine phosphatases CD45 and CD148, leading to downstream signal transduction (49, 50). Some studies have indicated that the distance between T-cell and APC is approximately 15 nm during the formation of an immunological synapse (51). This spatial distance excludes the phosphatases CD45 and CD148 from the immunological synapse because they have ectodomains that are longer than 15 nm (52, 53). Previous studies have demonstrated that the exclusion of CD45 from the cell–cell interphase is both necessary and sufficient for the formation of T-cell synapse (54, 55). The spatial distance between CAR T-cell and its target tumor cell may be equally important (Figure 3). However, it depends on entirely different structural elements, including the spatial structure of scFv, CAR position on the membrane, and location of the antigen on the target cell. Several studies have demonstrated that the identical epitope activates CAR T-cell with different levels of efficiency when expressed at different positions on the membrane. For example, Hombach et al. have explored the impact of a defined epitope position on the efficacy of CAR T-cell activation. They demonstrated that T-cell activation is more efficient when targeting the membrane proximal epitope than distal epitopes, indicating that the position of the targeted epitope has a major impact on the efficacy of T-cell activation (56). Hudecek et al. have also confirmed that the length and composition of IgG-derived extracellular spacer domains influence the function of CAR T-cells and that extracellular spacer domains lacking intrinsic signaling function are decisive in CAR design for an optimal *in vivo* activity (57). Thus, it might be an attractive strategy to enhance the sensitivity of CAR T-cell therapy by controlling the spatial distance in future research.

Previous studies have mainly focused on using exogenous activation elements, instead of intrinsic TCR, to remove MHC molecule restrictions. Recently, investigators developed a novel CAR T-like cell, known as bispecific T-cell engager (BiTE). This novel concept involves the use of a transgenic T-cell that can secrete T-cell-dependent bispecific antibodies, including two different scFv, one for tumor-specific antigens and another for T-cell specific antigens (often for TCR or CD3) (58). Because of its structure, the secreted scFv can link tumor cells with T-cells by acting as a bridge to activate intrinsic TCR/CD3 complex of BiTEs, but it is unknown whether CD4 or CD8 molecules of T-cells participate in this process because of the lack of MHC expression on tumor cells. A combination of endogenous TCR/CD3 and secreted scFv is sufficient to deliver signal 1, while intrinsic costimulatory molecules deliver signal 2. Luo et al. have developed BiTEs that are capable of secreting bispecific antibodies against CD3 and HER2, demonstrating an excellent antitumor effect. Interestingly, they have also highlighted that bispecific antibodies secreted by BiTEs affect the bystander T-cells not transfected with αHER2/CD3 RNA (59). However, the second activation signal for BiTEs, derived from intrinsic costimulatory agonists, has not yet been defined. Investigators need to administer an exogenous second activation signal to enhance the efficiency of BiTEs.

Another key factor influencing the sensitivity of CAR T-cell therapy is the T-cell intrinsic negative regulatory mechanism (60). For example, CAR T-cells successfully transferred into solid tumors often upregulated inhibitory factors, such as programmed death-1, cytotoxic T lymphocyte-associated antigen-4, T-cell immunoglobulin domain and mucin domain-3 (TIM3), and lymphocyte activation gene-3, that specifically bind to ligands on tumor cells to attenuate the antitumor efficacy. Combined immunotherapeutic strategy is promising for improving the sensitivity of CAR T-cell therapy (61, 62). Suarez et al. employed CAR T-cells targeting carbonic anhydrase IX (CAIX) expressed on metastatic clear cell renal cell carcinoma (ccRCC) in combination with programmed death ligand-1 (PD-L1) antibodies (63). In their design, CAR T-cells were engineered to secrete PD-L1 antibodies, which confirmed that local antibody delivery not only prevented T-cell exhaustion but also recruited NK cells into tumor sites. In a humanized mice model of ccRCC, tumor growth diminished five times and tumor weight reduced 50–80% compared with those in the anti-CAIX CAR T-cells alone. Moreover, increasing research groups have devoted their research into combinatorial immunotherapy besides combining with checkpoint inhibitors. For example, Junghans et al. have used anti-PSMA CAR T-cell combined with IL-2 for the treatment of prostate cancer, and they found that the clinical responses to CAR T-cells were restrained by low plasma IL-2. Therefore, a moderate dose of administered IL-2 is necessary to enhance CAR T-cell efficiency. This report also presented a unique example of the critical impact of the pharmacodynamics of drug–drug interactions on the efficacy of their coapplication (23). Curran et al. have established an approach to enhance CAR T-cells by expressing CD40 ligand (CD40L). T-cells modified to constitutively express CD40L (CD40L-modified T-cells) demonstrated an enhanced proliferation and secretion of pro-inflammatory cytokines *in vitro*. This research also showed that CD40L-modified CAR T-cells induce dendritic cell maturation and secretion of the pro-inflammatory cytokine IL-12 to enhance antitumor effects (64). Thus, in future experimental designs, investigators may consider infusing CAR T-cells with immune checkpoint inhibitors, cytokines, or other costimulatory molecules.

Furthermore, Nishio and Dotti developed a special combinational therapy that employed CAR T-cells combined with oncolytic viruses (OVs) that resulted in a remarkable antitumor effect compared with that using CAR T-cell or OV alone. Their research confirmed that OV supports T-cell function by remaining toxic to tumor cells without damaging or compromising CAR T-cell activities, even at high concentrations of OVs (65). They demonstrated that tumor cells infected by OVs become more susceptible to the lytic effects of CAR T-cells. In turn, the faster lysis of tumor cells facilitates the spread of the virus, which enhances CAR T-cells in solid tumor (66). Several studies have demonstrated that tumor-derived soluble factors and immunosuppressive immune cells in TME limit the sensitivity of CAR T-cells (67). These studies have suggested the importance of blocking the inhibitory factors of TME in CARs design (68). Within TME, various suppressive surveilling immune cells such as myeloid-derived suppressor cells (MDSCs), regulatory T-cells (Tregs), tumor-associated microphages (TAMs), or neutrophils (TANs) with M2 and N2 phenotypes present a barrier against antitumor immunity (69, 70). MDSCs, M2 TAMs, and N2 TANs are also well-known producers of transforming growth factor-β (TGF-β), IL-10, reactive oxygen/nitrogen species, nitric oxide synthase (NOS), and arginase (ARG) (71, 72). TGF-β is a critical cytokine in embryogenesis and tissue homeostasis. TGF-β can induce a large and diverse set of responses, ranging from the induction of tissue growth and morphogenesis in the embryo to the activation of cellular cytostatic and death processes in epithelial cells. However, in tumor tissues, increasing studies have confirmed that TGF-β prevents antitumor effects by inhibiting CD8 cytotoxic T lymphocytes and boosts tumor cell migration and proliferation (73). ARG and NOS are critical for l-arginine metabolism, which plays an important role in tumor immunity (74). The presence of l-arginine promotes T-cell effector function and memory T-cell differentiation. However, in several solid tumors, various suppressive surveilling immune cells overexpress one or both of these enzymes and lead to T-cell dysfunction because of arginine deficits within TME. Thus, the manipulation of tumor-derived soluble factors and immunosuppressive immune cell activity in tumor sites may enhance the efficacy of CAR T-cell therapies.

## Target Antigen Safety

Chimeric antigen receptor T-cells attack target cells by recognizing TAAs expressed on tumor cells. However, most TAAs are not only highly expressed on tumor cells but also shared with normal cells. Thus, the risk of “on target/off tumor” toxicity is a major obstacle for the development of CAR T-cell therapies for solid tumors. Several CAR T-cell therapies have resulted in life-threatening and fatal adverse events due to tumor lysis syndrome and cytokine storm. For example, Lamers et al. have evaluated the on-target toxicity of treatment of metastatic renal cell carcinoma with CAIX CAR-engineered T-cells. Common toxicity criteria grade 2–4 liver enzyme disturbances were observed in 4 of 12 patients at 1–2 × 10^9^ total cell dose, which can be prevented by pre-treatment with an anti-CAIX monoclonal antibody (35, 75). Morgan et al. have reported a serious adverse event in phase I clinical trials of an anti-HER2 CAR. In the third-generation (CD28.4-1BB.ζ) anti-HER2 CAR trial, a colon cancer patient with lung and liver metastases was intravenously administered with 1 × 10^10^ CAR T-cells. Within 15 min, the patient developed acute respiratory distress and died 5 days after treatment. They speculated that lung epithelial cells expressing HER2 at low levels were recognized by the administered cells and triggered a cytokine storm (34).

Ultimately, safety is closely dependent on specificity. In CAR design, we need to choose TAAs that are highly expressed on tumor cells but not expressed (or expressed at low levels) on normal cells as targets. Thus far, nearly all TAAs in the treatment of solid tumors have been expressed on normal tissues, particularly in bystander regions. The antitumor efficiency is associated with the dose of CAR T-cells, with high doses potentially increasing the risk of toxicity; therefore, it is difficult to balance safety and efficiency. Thus, researchers are advocating that suicide gene systems may ameliorate these toxicity profiles.

The herpes simplex virus–thymidine kinase (HSV-TK) suicide gene system has been most extensively tested in cell and gene therapy to eliminate the potential side effects of transduced cells (76–78). The HSV-TK gene has been successfully transferred into various cell lines to confer lethal sensitivity to the anti-herpes drug, ganciclovir, and its efficacy has been demonstrated both *in vitro* and *in vivo* (79). Another alternative suicide gene system is the inducible caspase 9 (iCasp9) gene, often used together with the small-molecule, AP1903 (80). The iCasp9 gene comprises an intracellular domain of the human caspase9 protein and a pro-apoptotic molecule fused to a drug-binding domain derived from human FK506-binding protein (81). This allows for dimerization and activation of apoptosis upon ligation with a dimerizer drug. This allows for dimerization and activation of apoptosis upon ligation with a dimerizer drug. The presence of AP1903 produces cross-linking of the drug-binding domains of the iCasp9 protein, which, in turn, dimerizes caspase 9 to activate the downstream executioner caspase 3 and results in cellular apoptosis (82). Diaconu et al. generated a novel CD19-specific CAR-modified T-cell (CD19.CAR Ts) selectively modulated by an iCasp9-based suicide gene. They demonstrated that the iCasp9 suicide gene depletes CD19.CAR T-cells in a dose-dependent manner in cases of cytokine release syndrome or complete deletion on demand, granting normal B cell reconstitution. In humanized mouse models, data also confirmed that low doses of AP1903 provide a specific containment for CD19.CAR T-cell expansion and cytokine release (83). Currently, CAR T-cells for solid tumors controlled by a specific safety switch are under study.

## Conclusion

Cancer presents a real threat to human health, and the advent of CAR T-cell represents the dawn of anti-cancer therapies, particularly for non-solid tumors. However, CAR T-cell therapy for solid tumors faces some challenges. The three main hurdles in the application of CAR T-cell therapies to solid tumors have been the identification of specific TAAs, limited trafficking of CAR T-cells to solid tumor sites, and immunosuppressive effect of TME. Here, we focus on CAR design to address the third problem of enhancing the specificity, sensitivity, and safety of CAR T-cells.

Several approaches to overcome the solid TME are discussed in this review. Researchers may combine CAR T-cell therapy with checkpoint inhibitors or design CARs targeting immune checkpoints (84). Investigators may also design CARs targeting TME concluding hypoxia, nutrient starvation, metabolism, stroma, and cytokine networks (85–89). For example, indoleamine 2,3-dioxygenase (IDO) is an intracellular enzyme expressed on tumor and myeloid cells, which blocks the proliferation and survival of CAR T-cells; therefore, it is feasible to develop a CAR targeting IDO or to combine CAR T-cells and IDO inhibitors for tumor treatment (90). Generating CARs that are capable of recognizing multiple antigens is also an effective alternative to address the hurdles of TAA identification. Researchers have also identified neoantigens specifically expressed on tumor cells as potential targets. Several groups have also demonstrated that the successful use of chemokine receptors matched with tumor cell chemokines can attract CAR T-cells to tumor sites (91).

The remarkable success of CAR T therapy in hematological malignancies has propelled the development of CAR T therapy in solid tumors (92, 93). A better understanding of tumorigenesis and tumor progression will drive advances of future cancer treatment and provide hope for preventing cancer.

## Author Contributions

YW, FL, JY, CZ, and YC conceived the theme of this review and wrote the manuscript.

## Conflict of Interest Statement

The authors did not receive any payment or services from a third party for any aspect of the submitted work. The authors also declare that they have no other relative affiliation or financial involvement with any organization or entity or other relationships/activities that may influence the submitted work.
